# Case Report: Testicular failure possibly associated with chronic use of methylphenidate

**DOI:** 10.12688/f1000research.5163.1

**Published:** 2014-09-02

**Authors:** Ranjith Ramasamy, Pranav Dadhich, Ashna Dhingra, Larry Lipshultz

**Affiliations:** 1 Department of Urology, Baylor College of Medicine, Houston, TX, 77030, USA

## Abstract

Methylphenidate is a commonly prescribed treatment for attention deficit hyperactivity disorder (ADHD). However, little is known about its adverse effects on the male reproductive system. We report a 20-year-old male patient whose chief complaint was of delayed puberty. He spoke in a high-pitched voice and complained of lack of body hair, impaired libido, inadequate erectile function, chronic fatigue, and low energy. He had been treated with methylphenidate as an infant and had continued treatment for 17 years. On examination, the patient was lean and visibly lacked facial or body hair. He further explained that he had never been able to grow underarm or facial hair and that he was often mistakenly considered a young teenager rather than a 20-year-old. The patient’s genitalia were categorized as Tanner Stage 2. Laboratory studies confirmed low serum follicle-stimulating hormone (FSH), luteinizing hormone (LH), and testosterone levels. The patient was given exogenous testosterone supplementation with pellets and human chorionic gonadotropin to maintain testicular size. After 4 months his symptoms improved and he demonstrated signs of puberty. Our goal is to further elucidate the possible impact of methylphenidate on the male reproductive system.

## Introduction

The use of methylphenidate (Ritalin ^®^) has surged over the past decade and is the hallmark therapy for treatment of attention deficit hyperactivity disorder (ADHD) ^1^. Moreover, the number of individuals diagnosed with ADHD has greatly increased within the past decade ^2^. Nevertheless, the long-term effects on various organ systems have not been fully evaluated.

Because of the increasing incidence of ADHD and the widespread use of methylphenidate in the pediatric population ^3^, it is important to determine whether methylphenidate has any adverse gonadotropic effects. Known adverse effects of methylphenidate include the drug’s impact on the cardiovascular system (high blood pressure, shortness of breath and irregular heart beat), on behavior, and mood (aggression, restlessness, hallucinations, unusual behavior, or motor tics); yet little information exists in the current literature regarding adverse effects of methylphenidate on the human reproductive system. Juvenile Rhesus monkeys treated with high doses of methylphenidate in a controlled experiment displayed a significant delay in testicular descent and were noted to have lower than normal serum testosterone levels ^4^. A parallel experiment was performed using male mice that were given increasing doses of methylphenidate. The mice treated with higher doses of methylphenidate experienced a significant decrease in body weight and reduction in Leydig cell count ^5^, indicating that serum testosterone levels and fertility were significantly reduced.

## Patient information

The patient, a 20-year-old Latino male, initially came to the clinic with a chief complaint of “delayed puberty”. In addition, he complained of his high-pitched voice, lack of libido, low energy level, chronic fatigue and poor erectile function. His height was similar to his friends of the same age although he was very lean and could not gain weight. The patient noted that on the basis of his physical appearance people often perceived him to be an adolescent, around the age of 12. He had never had facial or body hair, although he did have some pubic hair. The patient’s past medical history showed use of methylphenidate (dosage varied with age) for approximately 17 years with voluntary cessation a few years ago. The patient currently uses tobacco in the form of an electronic cigar and has been smoking for the past 8 years. He denied alcohol and drug usage. The patient is sexually active and engages in heterosexual sexual activity with no report of sexually transmitted disease. Family history was unremarkable.

## Clinical findings

On physical examination, the patient had neither underarm hair nor facial hair. He was 180cm and weighed 55kg. The patient’s genitalia were Tanner Stage II, (which is described to be occurring in males aged 8–15 years old and shows enlargement of the testes with pigmentation, minimal to no enlargement of the penis and long, downy hair with a variable pattern). The patient’s bilateral descended testes were 6cc in volume.

## Diagnostic assessment

Hormone testing revealed that the patient had a follicle-stimulating hormone (FSH) value of 3.0 mIU/ml (normal range is 4.0 – 10.0 mIU/ml), a luteinizing hormone (LH) value of 4 mIU/ml (normal range is 6.0 – 19.0 mIU/ml), and a serum total testosterone level of 120 ng/dl (normal values range from 200 to 1000 ng/dl). Semen analysis X 2 showed azoospermia both on initial examination and after use of pellet. The patient has a normal 46XY karyotype and no Y-chromosome microdeletion. The results of laboratory tests were consistent with the patient’s idiopathic testicular failure and warranted further exploration of the link between chronic methylphenidate use and effects on reproductive parameters.

## Therapeutic intervention

The patient was advised to begin testosterone supplementation. He chose to receive testosterone pellets (subcutaneous implantable testosterone). Testopel creates a depot of testosterone that is slowly released over a long period of time (~4–6 months). Because exogenous testosterone suppresses intrinsic testosterone synthesis, the patient was given 1500 IU human chorionic gonadotropin (hCG) therapy per week in order to maintain testicular size ^6^.

## Follow-up and outcomes

The patient was seen 4 months after his initial visit. He described a desirable increase in energy, increased libido, and better erectile function. Physical examination showed new facial and armpit hair, increased thickness of pubic hair, and maintenance of testicular size. Laboratory testing revealed low FSH (2.0 mIU/ml), and low LH (2 mIU/mL) levels, likely related to exogenous testosterone supplementation. On the other hand, the patient’s serum testosterone increased dramatically to 861 ng/dL. Overall, the patient was very enthusiastic about the progress associated with the therapy and underwent a second insertion of testosterone pellets. As for his fertility, a testicular biopsy for sperm retrieval with assisted reproduction may give him the best chance of fathering a biological child.

## Discussion

Although methylphenidate has been studied for many years, its effects on male gonadal function have only recently become a topic of interest among clinicians. The patient described in our case study seemed to exhibit characteristics related to the effects of chronic use of methylphenidate on development of human reproductive function.

Because the developmental changes that occurred in the human subject occurred over a number of years of treatment with methylphenidate, there is very little information about the patient’s condition and how it developed during that period of time. There is also little information for comparison with our observations because there is no much literature available regarding this topic.

Of the few previous studies on the effects of methylphenidate on male gonadal function, one shows results obtained using Wistar rats tested with increasing amounts of methylphenidate. The data show an increase in abnormalities in sperm and a decrease in testicular volume ^7^. Another study using Wistar rats showed that increasing levels of methylphenidate led to increased p53 expression and apoptosis of germ cells ^8^.

The study involving rats reveals similarities to the case study in question. The case reported describes a possible association between methylphenidate use and testicular failure. The rats were given increasing doses of the drug over a long period of time; our patient was also taking the drug to treat ADHD for a very long time (~14 years). However, while the rats were monitored from the beginning of the drug treatment, the subject in the case study has only recently come under observation after 14 years of methylphenidate use.

Another of the few existing studies was reported in the *Proceedings of the National Academy of Sciences*. It was conducted on Rhesus monkeys treated with doses of methylphenidate at designed to mimic the amounts given to human ADHD patients ^4^. This was one of the first recorded studies displaying altered testicular function in primates following methylphenidate treatment. Over a 40 month time span, the monkeys displayed signs of delayed puberty, including impaired testicular descent and smaller-sized testicles, as well as lower testosterone levels ^9^. As noted above, delayed puberty was also observed in our patient, as demonstrated by the Tanner staging and the low testosterone levels in serum. Previous human research yielded similar results. Studies conducted in adolescent boys showed delays in puberty ^10^. The drug of first choice was methylphenidate, although some subjects were given dexamphetamine. Subjects receiving either methylphenidate or dexamphetamine showed lower weight and BMIs coupled with delayed pubertal development ^10^. Although the testicular damage induced by methylphenidate is well recognized, the precise mechanisms underlying its toxicity to the testes remains unclear. It is tempting to speculate that methylphenidate could affect the alpha and beta-adrenergic receptors expressed in the testis ^6^.

This case report, however, specifically pinpoints methylphenidate and shows an unusual prescription practice for the patient involved. In this case, methylphenidate was started from a very young age compared to normal prescription practices for ADHD. The unknown effects of methylphenidate are currently being studied, but as can be seen, one should exercise caution and patients should be followed closely when prescribing methylphenidate.

## Consent

Written informed consent to publish this case report was obtained from the patient.
