# Supplementary material for: Case Report: Testicular failure possibly associated with chronic use of methylphenidate
Source: F1000Res. 2014 Sep 2;3:207. [Version 1] doi: 10.12688/f1000research.5163.1 (PMC4215747; doi:10.12688/f1000research.5163.1)
Supplement: Peer review history

# Peer review: Referee response for version 1

**DOI:** 10.5256/f1000research.5507.r6011

**Published:** 2014-09-11

**Authors:** Jay Sandlow, Christopher Deibert

**Affiliations:** 1 Department of Urology, Medical College of Wisconsin, Milwaukee, USA

SUMMARY OF STUDY  

The authors present a case report in which there is an association between chronic methylphenidate use from a very young age and delayed puberty and reproductive growth. The use of the term “testicular failure” is a misnomer. His low gonadotropins make this secondary testicular failure. There is no evidence that he has primary testicular failure, as the authors do not present any data to show lack of response to gonadotropins. At best, this may be an association. 

SPECIFIC COMMENTS FOR AUTHORS

Introduction

- The introduction should conclude with a restatement of the goal or thesis of the manuscript.  It currently ends describing mice fertility.

Case

- Please clarify for the semen analysis “after use of pellet”. Do the authors mean after the examination of the spun down SA pellet? If so please rephrase this.
- Was delayed puberty ever ruled out in this patient? Was he examined by a pediatric endocrinologist?
- Why was the patient not started on HCG alone to see what his testicular response would be?  By giving him exogenous testosterone, his gonadotropins will remain low and his spermatogenesis suppressed.
- In follow up, please describe more clearly the upgrading in his Tanner stage

Discussion

- The second sentence of the discussion is much too strong. To this point, the authors have provided no compelling evidence of the relationship between chronic Ritalin use and reproductive function. Only midway through the discussion do the authors describe this more as an association than a causal mechanism. Clarify at the outset that there is no human data suggesting this relationship currently available.
- For the rat studies, did these rats have exposure prior to adolescence and signs of maturation?  Only in this way can the rats possibly be compared to this human and serve as a more direct causal possibility.
- Reference 10, as the only human study cited, should be explored more than the rat and monkey studies. It should probably also be described earlier in this case report.
- The authors should state whether or not there is data from the methylphenidate approval trials that demonstrates suggestion of delayed puberty or delayed development among prepubertal boys taking this medication.
- In the concluding paragraph, the authors again should consider tempering their comments, that this is at most an association and not a direct causal relationship.

We have read this submission. We believe that we have an appropriate level of expertise to confirm that it is of an acceptable scientific standard, however we have significant reservations, as outlined above.

# Peer review: Referee response for version 1

**DOI:** 10.5256/f1000research.5507.r6064

**Published:** 2014-09-08

**Authors:** Matthew Coward

**Affiliations:** 1 Department of Urology, University of North Carolina, Chapel Hill, USA

This is a well written case report of an unrecognized, but only possible, association between use of Ritalin ^® ^and delayed puberty. The discussion offers a very nice commentary on the available literature, and this paper overall generates appropriate consideration for this potential serious adverse effect of methylphenidate.  The patient reported had almost lifelong use of methylphenidate throughout childhood and adolescence, but stopped several years prior to presenting with delayed puberty.  Another reviewer made several very important comments that I was going to make, so I will not repeat them.  A few additional considerations to improve this very nice manuscript are discussed below:

- This patient's particular situation was unusual in that he presented with normal gonadotropins along with hypogonadism, and this should be discussed.  Delayed puberty is not typically a testicular problem, but rather one with the HPG axis.  The hypothesis would be that the methylphenidate has a direct suppressive effect at the level of the testicles.
- The combined use of exogenous testosterone and hCG has been reported by Hsieh *et al.* to maintain spermatogenesis in hypogonadal men, but the combination has not been previously reported to initiate puberty.  It certainly would be the most physiologic manner to initiate puberty, and this point could be highlighted.
- The options for treatment should be discussed.  These can include several options such as exogenous testosterone, high dose hCG, or a combination of the two using a lower dose of hCG which was ultimately chosen.
- Please include the dose of Testopel used.
- The androgenization of the patient is noted through the use a exogenous testosterone.  An improvement in testicular function is not documented by the authors.  This can include post-therapy changes in testicular volume, or more accurately a post-therapy semen analysis.  
- In the Follow-up and Outcomes section, the sentence beginning, "As for his fertility..." falls flat with mere speculation.  A brief discussion of the potential benefit from ultimately stopping exogenous testosterone and reversing its effects with higher dose hCG is warranted, as it is unclear from this report that the patient has irreversible testicular atrophy and hypofunction that would result in long-term non-obstructive azoospermia requiring testicular sperm extraction.  A simple follow-up semen analysis would be the most accurate way to update the manuscript and frame this discussion.

I have read this submission. I believe that I have an appropriate level of expertise to confirm that it is of an acceptable scientific standard.

# Peer review: Referee response for version 1

**DOI:** 10.5256/f1000research.5507.r6010

**Published:** 2014-09-03

**Authors:** Wayland Hsiao

**Affiliations:** 1 Department of Urology, Emory University, Atlanta, USA

Very interesting case report of a young man with delayed puberty and primary testicular failure, with no identifiable known cause. The only thing suspicious on their history was long-term use of methylphenidate, with no identifiable genetic cause found otherwise.

The paper talks about the treatment of this patient with testosterone replacement and then reviews the limited animal data on methylphenidate and its effects on spermatogenesis.  Of course, as with any case report and retrospective review, it is difficult to say for certain whether this is associated or there is another unknown confounding factor involved, so the authors suggestion that reproductive harm has to be taken into account before giving methylphenidate to children is premature at best.

However, this case does highlight the real dearth of information and studies we have on medications that are widely used and their effects on sperm production and reproductive potential. While this drug is so widely prescribed we only have a number of rat studies and a primate study, all of which have limitations in their ability to be generalized to human results.

As a result, this is a great case report for generating ideas, and raising the idea that maybe some things we commonly prescribe can cause reproductive effects we are unsure of, but to say there is an association, and then say that methylphenidate should be prescribed less because of this is a bit of a stretch.

I have read this submission. I believe that I have an appropriate level of expertise to confirm that it is of an acceptable scientific standard, however I have significant reservations, as outlined above.

# Response

**Published:** 2014-09-03

**Authors:** Ranjith Ramasamy

**Affiliations:** Baylor College of Medicine, USA

We agree with the reviewer that the association between chronic ritalin use and testicular failure is not direct. The case report is at best hypothesis generating but given some of the strong evidences available in animal studies, the practitioner should be cognizant of a "possible" association between chronic ritalin use and infertility and delayed puberty.
